# Supplementary material for: A data integration framework for spatial interpolation of temperature observations using climate model data
Source: PeerJ. 2023 Jan 10;11:e14519. doi: 10.7717/peerj.14519 (PMC9838203; doi:10.7717/peerj.14519)
Supplement: Supplemental Information 1 [file peerj-11-14519-s001.pdf]

All the data, code and supplementary material are available and can be accessed at Zenodo at:

https://doi.org/10.5281/zenodo.7049824.
